# A Unique Sugar l-Perosamine (4-Amino-4,6-dideoxy-l-mannose) Is a Compound Building Two O-Chain Polysaccharides in the Lipopolysaccharide of *Aeromonas hydrophila* Strain JCM 3968, Serogroup O6

**DOI:** 10.3390/md17050254

**Published:** 2019-04-28

**Authors:** Katarzyna Dworaczek, Maria Kurzylewska, Magdalena A. Karaś, Monika Janczarek, Agnieszka Pękala-Safińska, Anna Turska-Szewczuk

**Affiliations:** 1 Department of Genetics and Microbiology, Maria Curie-Skłodowska University in Lublin, Akademicka 19, Lublin 20-033, Poland; pakiet.kat@gmail.com (K.D.); mariakurzylewska@wp.pl (M.K.); magdalena.karas@poczta.umcs.lublin.pl (M.A.K.); mon.jan@poczta.umcs.lublin.pl (M.J.); 2 Department of Fish Diseases, National Veterinary Research Institute, Partyzantów 57, Puławy 24-100, Poland; a.pekala@piwet.pulawy.pl

**Keywords:** *Aeromonas hydrophila*, lipopolysaccharide (LPS), endotoxin, structure elucidation, O-antigen, O-polysaccharide, l-perosamine, l-Rha4NAc, NMR spectroscopy, MALDI-TOF mass spectrometry

## Abstract

Lipopolysaccharide (LPS) is the major glycolipid and virulence factor of Gram-negative bacteria, including *Aeromonas* spp. The O-specific polysaccharide (O-PS, O-chain, O-antigen), i.e., the surface-exposed part of LPS, which is a hetero- or homopolysaccharide, determines the serospecificity of bacterial strains. Here, chemical analyses, mass spectrometry, and ^1^H and ^13^C NMR spectroscopy techniques were employed to study the O-PS of *Aeromonas hydrophila* strain JCM 3968, serogroup O6. MALDI-TOF mass spectrometry revealed that the LPS of *A. hydrophila* JCM 3968 has a hexaacylated lipid A with conserved architecture of the backbone and a core oligosaccharide composed of Hep_6_Hex_1_HexN_1_HexNAc_1_Kdo_1_P_1_. To liberate the O-antigen, LPS was subjected to mild acid hydrolysis followed by gel-permeation-chromatography and revealed two O-polysaccharides that were found to contain a unique sugar 4-amino-4,6-dideoxy-l-mannose (*N*-acetyl-l-perosamine, l-Rha*p*4NAc), which may further determine the specificity of the serogroup. The first O-polysaccharide (O-PS1) was built up of trisaccharide repeating units composed of one α-d-Gal*p*NAc and two α-l-Rha*p*4NAc residues, whereas the other one, O-PS2, is an α1→2 linked homopolymer of l-Rha*p*4NAc. The following structures of the O-polysaccharides were established:

O-PS1 →3)-α-l-Rha*p*4NAc-(1→4)-α-d-Gal*p*NAc-(1→3)-α-l-Rha*p*4NAc-(1→
O-PS2 →2)-α-l-Rha*p*4NAc-(1→

The present paper is the first work that reveals the occurrence of perosamine in the l-configuration as a component of bacterial O-chain polysaccharides.

## 1. Introduction

*Aeromonas* bacteria are ubiquitous in aquatic ecosystems, such as seawater, freshwater, and estuarine and coastal waters. They were isolated from chlorinated potable water as well as raw and processed food. *Aeromonas* are either mesophilic motile or psychrophilic non-motile Gram-negative rods [1,2,3]. They have also been identified as components of the gut microbiome in fish, amphibians, and reptiles. In environmental stress conditions (overcrowding, poor water quality, organic pollution, and hypoxia), these opportunistic bacteria may cause various diseases in poikilothermic animals [4,5]. The species *Aeromonas hydrophila*, *Aeromonas salmonicida*, *Aeromonas caviae*, *Aeromonas veronii*, *Aeromonas sobria*, and *Aeromonas bestiarum* have been described as important pathogens in fish. They may cause chronic disease with skin ulceration or acute systemic infection, referred to as motile aeromonad septicemia (MAS), as well as other pathological lesions [5,6,7]. Water and food may be a source of *Aeromonas*-origin infections in humans; immunocompromised patients and children are especially vulnerable. Of the 21 species classified on the basis of DNA-DNA hybridization, *A. hydrophila*, *A*. *caviae*, and *A*. *veronii* bt. *sobria* are the most common species known to cause human diseases. They have been recognized as agents of both gastrointestinal diseases and life-threatening extraintestinal infections (wound infections, urinary tract infections and septicemia, occasionally meningitis and peritonitis) [5,8,9]. 

Extracellularly secreted products of *Aeromonas* metabolism, including hemolysins, cytotonic and cytotoxic enterotoxins, proteases, lipases, and leucocidins, have been suggested as possible contributory factors in the pathogenesis of these bacteria [5,10]. Amongst these, the type II secretion system (secretion of enterotoxin - Act) and the type III secretion system (T3SS) seem to be leading [11]. Moreover, in Gram-negative bacteria, e.g., *Aeromonas* rods, cell-surface components including outer membrane (OM) proteins, lipopolysaccharide (LPS), S-layer, polar flagella, and pili (type IV and bundle-forming pili) have been implicated as virulence factors [12,13,14,15,16]. 

LPS, the major glycolipid of the OM, consists of three covalently linked regions: Lipid A (a biological center of endotoxin), the core oligosaccharide (core region), and the O-specific polysaccharide (O-chain, O-antigen), which is a hetero- or homopolysaccharide regarded as the outer cell antigen determining the serospecificity of bacterial strains [17]. High Molecular Weight (HMW) smooth S-type LPS with O-polysaccharide (O-PS) is essential for the adhesion of microorganisms to host tissue and is necessary during infection events, where it protects them from complement-mediated killing and antimicrobial peptides [5]. The structural diversity of the O-chain seems to serve this function, which contributes to the wide variety of antigenic types between species and even strains of Gram-negative bacteria, determining the specificity of each bacterial serotype. On the other hand, serological cross-reactivity sometimes indicates similarities or identities between O-antigens [17,18]. Although it was not clearly evidenced which structural determinants are the most important for virulence, it was found that some O serotypes are more frequently associated with certain infections. 

*Aeromonas* strains are serologically heterogeneous and, according to the first useful NIH scheme (National Institute of Health, Japan) proposed by Sakazaki and Shimada [19], they are classified into 44 serogroups based on O-antigens, which can be further extended to 97 O serogroups, after the inclusion of provisional new serogroups (PG) described by other investigators [20]. As demonstrated in some previous studies, the distribution of the serogroups of *Aeromonas* strains may be related to geographic localization. Studies have demonstrated that *Aeromonas* strains belonging to serogroups O11, O16, O18, and O34 (Sakazaki and Shimada scheme) are associated with most cases of bacteremia, implying the O-PS serotypes are relevant in the pathogenesis of some systemic diseases [5]. Geographical differences in the distribution of serogroups of *Aeromonas* species are associated with outbreaks of septicemia in fish as well. The strains of serogroups O11, O16, O34, and O14 have been related to motile aeromonas septicemia (MAS) incidences in rainbow trout. In turn, strains belonging to O14 have been identified as pathogens for European eels [21]. As shown by Kozińska and Pękala [22], the majority of isolates pathogenic to carp in Polish aquacultures represented serogroups O3, O6, O41, PGO4, and PGO6, whereas serogroups O11, O16, O18, O33, PGO1, and PGO2 dominated among both carp and trout isolates. Until now, the structure of a few *Aeromonas* O-antigen polysaccharides that tag strains as belonging to serogroups O11, O14, O34, O16, and O18 were established [23,24,25,26,27,28]. Moreover, the chemical composition of the O-antigens of *A. salmonicida* subsp. *salmonicida* typical strains A449 and 80204 isolated from salmonids and atypical ones [29,30], *A. caviae* strain ATCC 15468 [31], *A. sobria*, and *A. bestiarum*, have been characterized as well [32,33,34]. 

The O-chain polysaccharide in a majority of *Aeromonas* strains has a heteropolymeric structure with repeating units, which contain mostly 6-deoxyhexoses, hexoses, aminohexoses, and amino 6-deoxyhexoses, frequently decorated with acidic non-carbohydrate groups e.g., *O*-acetyl, *N*-acetyl, and 3-hydroxy butyryl [29,34]. 

In the present study, we have shown the structural characterization of LPS including an O-specific polysaccharide isolated from *A. hydrophila* strain JCM 3968, which is a reference strain for serogroup O6. We have demonstrated that the O-chain, which is composed of two structurally different O-polysaccharides, contains a unique sugar 4-acetamido-4,6-dideoxy-l-mannose (l-Rha4NAc), which may thus determine the specificity of serogroup O6. To the best of our knowledge, this is the first paper describing the occurrence of a 4-acetamido-4,6-dideoxy-mannose (perosamine) in the absolute configuration l (l-perosamine) as a component of the bacterial O-specific polysaccharide, O-antigen. 

## 2. Results

### 2.1. Bacterial Cultivation, Isolation of LPS, and SDS-PAGE Study 

**Figure 1:**
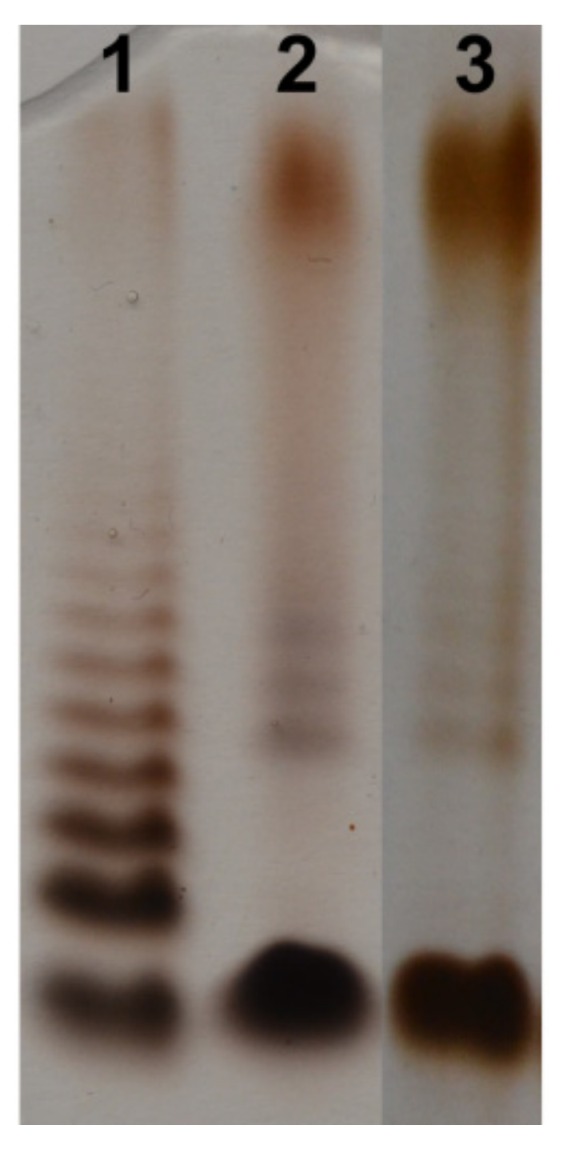
Silver-stained SDS-PAGE of the LPS of *A. hydrophila* strain JCM 3968, O6 (lane 2, and 3, 2 and 3 μg, respectively) and *Salmonella enterica* sv. Typhimurium (Sigma-Aldrich, St. Louis, MO, USA) as reference (lane 1, 2 μg).

Bacterial cells of *Aeromonas hydrophila* strain JCM 3968 were extracted with hot aq 45% phenol [35], and LPS species were harvested from the phenol phase in a yield of 4.8% of the bacterial cell mass. SDS-PAGE analysis of the LPS followed by silver staining showed a pattern typical for LPS isolated from smooth bacterial cells (Figure 1) with the content of both a slow migrating S-LPS and a fast migrating rough R-LPS glycoforms. 

### 2.2. Chemical and Mass Spectrometry Analyses of LPS

Compositional analysis of the degraded polysaccharide (dgPS) liberated from the phenol-soluble LPS was performed by GLC-MS of alditol acetates. It showed the presence of d-glucose (d-Glc), d-galactose (d-Gal), 2-amino-2-deoxy-d-glucose (d-GlcN), d-*glycero*-d-*manno*-heptose (d,d-Hep) and l-*glycero*-d-*manno*-heptose (l,d-Hep) in a ratio of 1.0:1.2:1.3:3.0:2.8 as the core oligosaccharide components. The chemical analysis also revealed 6-deoxymannose (Rha), 2-*O*Me-4-amino-4,6-dideoxyhexose, 4-amino-4,6-dideoxyhexose (identified as Rha4N, see Section 2.3), and GalNAc. These two latter sugars were identified as the main components of the O-PS part (see Section 2.3.). Kdo (3-deoxy-d-*manno*-2-octulosonic acid)—the only acidic sugar—was found in the LPS after HF (hydrofluoric acid) treatment of the LPS, which suggested its phosphorylation [36,37]. The GLC-MS analysis of fatty acids as methyl esters and *O*-TMS derivatives revealed that 3-hydroxytetradecanoic [14:0(3-OH)] and dodecanoic (12:0) acids were the most abundant species. GlcN was identified as the sugar component of the lipid A. 

**Figure 2:**
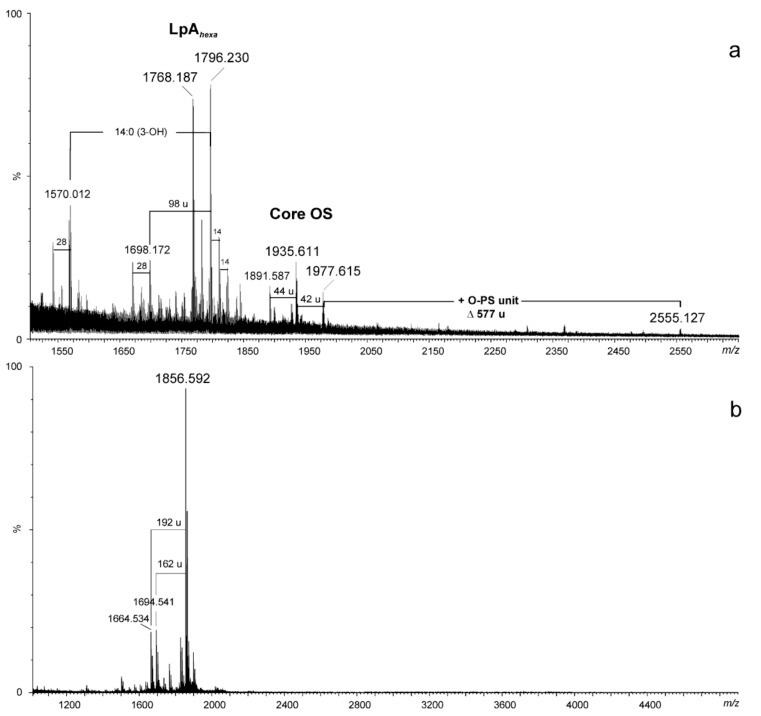
MALDI-TOF mass spectra (negative ion mode) of the LPS (**a**) and the core oligosaccharide (**b**) of *A. hydrophila* JCM 3968. The notations indicate: 14 u and 28 u – the differences in CH_2_ and (CH_2_)_2_ in the fatty acid chains, respectively; 42 u - loss of ketene; 44 u – loss of CO_2_; 98 u – loss of phosphate plus water; 162 u - loss of hexose, 192 u - loss of heptose; LpA*_hexa_* - hexaacylated lipid A.

The negative ion matrix-assisted laser desorption/ionization time-of flight (MALDI-TOF) mass spectrum of the *A. hydrophila* JCM 3968 lipopolysaccharide (Figure 2a) showed the most intensive signals in the *m/z* range 1600–2000. These were Y- and B-type fragments [38] originating from cleavage of the labile ketosidic linkage between lipid A and Kdo of the core oligosaccharide in the LPS molecule as a consequence of in-source fragmentation [28]. The ions at *m/z* 1768.18 and 1796.23 were Y-type fragments of hexaacylated lipid A species. The latter one represented a variant of lipid A, where the diglucosaminyl backbone bisphosphorylated at O-1 and O-4’ is substituted by three, instead of four, 3-hydroxytetradecanoic acids, 14:0(3-OH), one 3-hydroxy*iso*pentadecanoic acid, *i*15:0(3-OH), and two dodecanoic acids, 12:0. The composition of the ions is shown in Table 1. In turn, the ions at *m/z* 1977.6 and 1935.6 were assigned to the core oligosaccharide (B-type) with the following composition: Hep_6_Hex_1_HexN_2_Kdo*_anh_*P; the mass difference between the ions of 42 amu corresponded to oligosaccharides containing an acetylated and non-acetylated variants of HexN, respectively. In addition, a further B-type ion was observed at *m/z* 2555.127, which was attributed to the core oligosaccharide (OS) with one O-antigen repeating unit attached. The mass difference of 577 amu, corresponding to the following composition: (6dHexNAc)_2_HexNAc-H_2_O (calcd monoisotopic mass: 577.24), was in full agreement with the chemical structure of the O-PS1 repeating unit determined by NMR (see Section 2.3.). 

The negative ion mass spectrum recorded for the core oligosaccharide, which was liberated from LPS after mild acid hydrolysis and separation by gel-permeation-chromatography, GPC, showed the major ion at *m/z* 1856.59, corresponding to the core decasaccharide with the composition Hep_6_Hex_2_HexN_1_Kdo*_anh_* (calcd monoisotopic mass, = 1857.613 amu, calcd mass of deprotonated molecule = 1856.606 amu) (Figure 2b, Table 1). The methylation analysis of the *N*-acetylated oligosaccharide fraction, followed by full acid hydrolysis and GLC-MS of partially methylated monosaccharides as acetylated alditols-1-*d*, revealed terminal Gal, terminal GlcN, 6-substituted Glc, terminal d,d-Hep, terminal l,d-Hep, and 4,6-disubstituted d,d-Hep, 7-substituted, 2-substituted, and 3,4,6-trisubstituted l,d-Hep. A similar composition and glycosylation pattern of the monosaccharides as well as the measured molecular mass of the core oligosaccharide was shown for the LPS core OS of *A. hydrophila* AH-901, i.e., a rough mutant strain derived from *A. hydrophila* AH-3 [36]. 

In turn, the identification of the 3-substituted GalNAc detected among permethylated alditol acetates of the dgPS and the composition of the core oligosaccharide indicating the presence of *N*-acetylated hexosamine rather than a hexose residue (established based on the mass spectrometry data of intact LPS) might suggest that the O-antigen in the LPS of *A. hydrophila* strain JCM 3968 is linked to the GalNAc as in *A. salmonicida* subsp. *salmonicida* A450 LPS [39]. 

### 2.3. Structural Studies of O-polysaccharide (O-PS)

The O-PS was released from phenol-soluble LPS by mild-acid degradation followed by gel-permeation-chromatography (GPC) on Sephadex G-50 fine to give a high-molecular-mass O-polysaccharide with the yield of 31% of the LPS mass. GLC-MS sugar analysis of alditol acetates obtained after full acid hydrolysis of the O-PS showed the presence of 4-amino-4,6-dideoxyhexose identified as 4-amino-4,6-dideoxymannose (Rha4N) by comparison with an authentic sample from the polysaccharide of *Citrobacter gillenii* O9a,9b (strain PCM 1537) [40] and galactosamine (GalN) in a peak area ratio ~ 4.2:1. Among amino sugar derivatives, a small amount of 2-*O*-methyl-4-amino-4,6-dideoxyhexitol acetate (Rha4N2Me) was found as well. Other compounds detected in the GLC chromatogram of the *A. hydrophila* 3968 O-PS, i.e., GlcN and two heptose isomers (d,d-Hep and l,d-Hep), represented the core oligosaccharide sugars. 

Determination of the absolute configurations of the monosaccharides by GLC of the acetylated (*S*)- and (*SR*)-2-octyl glycosides [41] showed that Rha4N had the l configuration and GalN had the d configuration. A sample from the polysaccharide of *Citrobacter gillenii* O9a,9b [40] was used as a reference standard of d-Rha4N. The l configuration of Rha4N was also confirmed by the analysis of glycosylation effects in the ^13^C NMR spectrum of the O-PS (see below).

The methylation analysis of the O-PS by GLC-MS of partially methylated alditol acetates resulted in identification of 4,6-dideoxy-3-*O*-methyl-4-(*N*-methyl)acetamidohexose (derived from 2-substituted Rha4N), 4,6-dideoxy-2-*O*-methyl-4-(*N*-methyl)acetamidohexose (derived from 3-substituted Rha4N), and 2-deoxy-3,6-di-*O*-methyl-2-(*N*-methyl)acetamidohexose (derived from 4-substituted GalN). Additionally, the CD_3_-methylation indicated a small amount of 4,6-dideoxy-2-*O*-methyl-4-(*N*-CD_3_)-acetamidohexose (derived from 3-substituted 2-*O*Me Rha4N). The *EI* mass spectrum of 4,6-dideoxy-2-*O*-methyl-4-(*N*-methyl)acetamidomannose was characterized by the presence of intense ion peaks at *m/z* 118 (C-1 ÷ C2 fragment), 275 (C-1 ÷ C4 fragment), and 172 (C-4 ÷ C6 fragment), and allowed distinguishing this derivative from 4,6-dideoxy-3-*O*-methyl-4-(*N*-methyl)acetamidomannose, whose *EI* mass spectrum contained ions at *m/z* 190, 275, and 172 characteristic of the C-1 ÷ C3, C-1 ÷ C4, and C-4 ÷ C6 primary fragments, respectively. 

**Figure 3:**
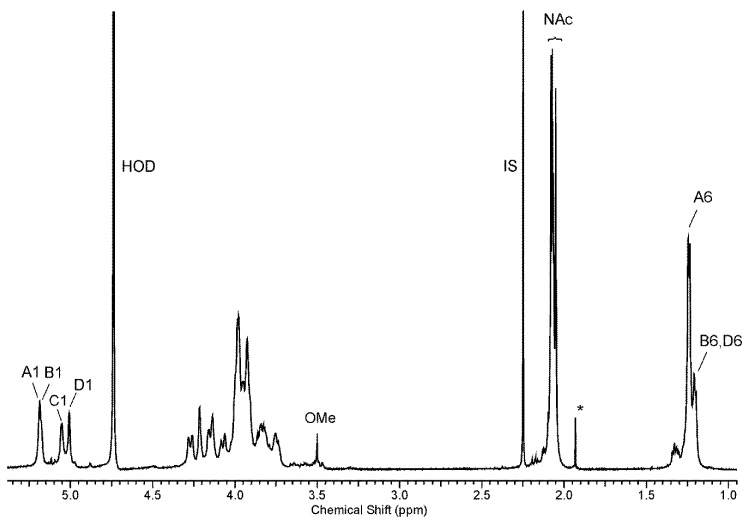
^1^H NMR spectrum of the O-PS of *A. hydrophila* JCM 3968, serotype O6. The spectrum was recorded in D_2_O at 32 °C at 500 MHz. Capital letters and Arabic numerals refer to atoms in the sugar residues denoted as shown in Table 2. NAc - *N*-acetyl groups, IS – acetone as an internal standard (δ_H_ 2.225), OMe – *O*-methyl group, asterisk – free acetic acid.

The low-field region of the ^1^H NMR spectrum of the O-polysaccharide (Figure 3) contained four major signals for anomeric protons at δ 5.17, 5.16, 5.04, and 5.0. The high-field region of the spectrum included signals for *N*-acetyl groups at δ 2.05–2.06, CH_3_-C groups of 6-deoxy sugars in the range of δ 1.19–1.23, and a less intense signal of an *O*-methyl group at δ 3.5; some of the signals overlapped. The ^1^H and ^13^C resonances of the O-PS of *A. hydrophila* JCM 3968, O6 were assigned using 2D homonuclear ^1^H,^1^H DQF-COSY, TOCSY, NOESY, heteronuclear ^1^H,^13^C HSQC, and HMBC experiments. The ^1^H and ^13^C NMR data are collected in Table 2. 

**Figure 4:**
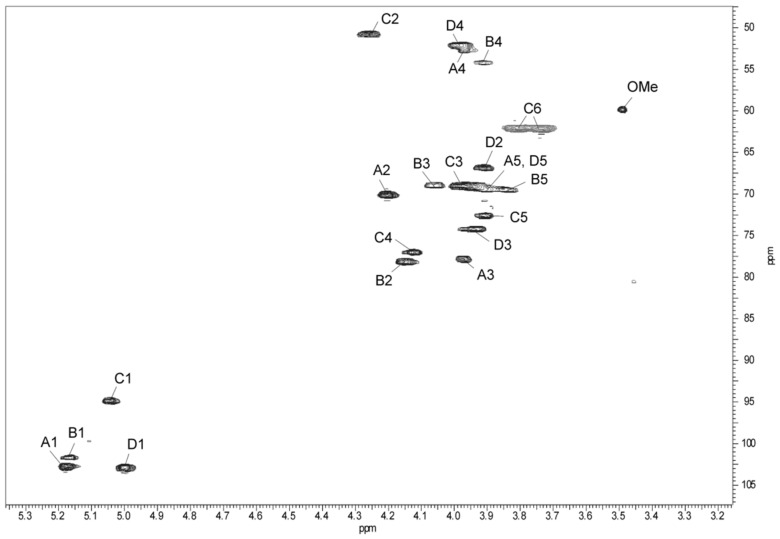
Part of a ^1^H,^13^C HSQC spectrum (500 × 125 MHz) of the O-PS of *A. hydrophila* JCM 3968, serotype O6. The spectrum was recorded at 32 °C in D_2_O as a solvent. Capital letters and Arabic numerals refer to atoms in sugar residues denoted as shown in Table 2. OMe - *O*-methyl group of Rha4N2Me, chemical shifts for *O*Me are δ_H_ 3.5 and δ_C_ 60.0.

The ^1^H,^13^C HSQC spectrum (Figure 4) showed four intense correlation signals at δ 3.91/54.3, 3.97/52.5, 3.98/52.3, and 4.26/50.9 of the proton at the nitrogen-bearing carbon to the corresponding carbon and indicated that the O-PS is built up of *N*-acetamido sugars. 

The ^1^H,^1^H TOCSY, and DQF-COSY spectra revealed four major spin systems for monosaccharide residues, which were labelled **A**–**D** in the order of the decreasing chemical shifts of their anomeric protons. The **A**, **B**, and **D** spin systems (H-1/C-1 cross-peaks at δ 5.17/103.0, 5.16/101.7, and 5.0/103.0, respectively) were typical of *manno-*pyranose, as indicated by low vicinal coupling constants ^3^*J*_1,2_ (~2 Hz), and high ^3^*J*_3,4_ (8.5 Hz), ^3^*J*_4,5_ (9 Hz), and ^3^*J*_5,6_ (6 Hz) values. They were assigned to Rha4NAc residues based on high-field positions of their H-6 and C-6 resonances and taking into account nitrogen-bearing carbons. 

In the TOCSY spectrum, starting from the H-2 proton signal, cross-peaks with H-2-H-6 for Rha4NAc **B** as well as Rha4NAc **A** and Rha4NAc **D** were visible; however, some signals of the two latter residues overlapped. The ^1^H,^1^H COSY spectrum allowed unambiguous differentiation between protons within the spin system **B** and only partly resolved the cross-peaks for the Rha4NAc **A** and Rha4NAc **D**. The difficulties in the assignment of the H-3, H-4, and H-5 of Rha4NAc **A** and **D** were overcome in the ^1^H,^13^C HMBC and ^1^H, ^13^C HSQC experiments. 

The ^13^C NMR resonances of Rha4NAc **A** were assigned by the long-range H-6/C-4 and H-6/C-5 correlations at δ 1.23/52.5 and 1.23/69.4, and then the C-4/H-3 correlation at δ 52.5/3.97 in the ^1^H,^13^C HMBC spectrum. In the ^1^H,^13^C HSQC spectrum, the cross-peak of the proton at the nitrogen-bearing carbon to the corresponding carbon at δ 3.97/52.5 was assigned to the H-4/C-4 correlation of Rha4NAc **A**. Similar long-range H-6/C-4 and H-6/C-5 correlations were searched during identification of H-4 and H-5 proton signals of Rha4NAc **D**. For the latter residue, correlations of the anomeric proton of Rha4NAc **D** with carbons C-2, C-3, and C-5 were found in the ^1^H,^13^C HMBC spectrum, and then the proton resonances were assigned from the ^1^H,^13^C HSQC spectrum. The chemical shifts of the C-3 signals of Rha4NAc **A** and **D** were identified after consideration of the methylation analysis data and the glycosylation effects on the ^13^C NMR resonances. The fifth spin system at δ_H/C_ 5.11/100.0, with much less intense correlation signals and not completely resolved resonances, was assigned to the Rha4N2Me residue. 

In the TOCSY spectrum, correlations for the *galacto* configuration of the spin system **C** were visible between H-1 and H-2,H-3,H-4, and other proton signals were assigned by connectivities observed in the NOESY (H-3/H-5; H4/H-6) and COSY (H-6/H-5) spectra. In the ^1^H,^13^C HSQC spectrum, the correlations of H-2 at δ 4.26 with a nitrogen-bearing carbon at δ 50.9 and H-6 protons with C-6 at δ 3.74;3.81/62.2 completed identification of the GalNAc residue **C**. 

**Figure 5:**
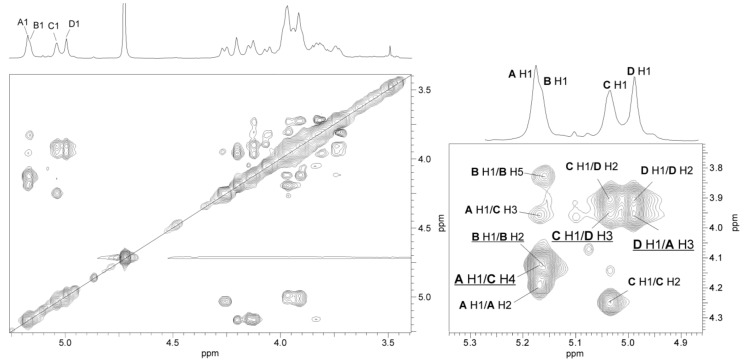
Parts of ^1^H,^1^H NOESY and ^1^H NMR (insert) spectra of the O-PS of *A. hydrophila* JCM 3968, serotype O6. The map shows NOE contacts between anomeric protons and protons at the glycosidic linkages (underlined). Some other H/H correlations are depicted as well. Capital letters and Arabic numerals refer to atoms in the sugars denoted as shown in Table 2.

The relatively small ^3^*J*_1,2_ coupling constant value of <4 Hz indicated that GalNAc is α-linked. This conclusion was confirmed by relatively high-filed position of the C-5 signal at δ 72.7, compared with δ 71.7 and δ 76.4 for α-GalNAc and β-GalNAc, respectively [42]. The α-configuration of all Rha4NAc residues was inferred by a relatively high-field position of the C-5 signals at δ 69.4–69.6 for the residues in the O-polysaccharides, compared with δ 72.4 for β-Rha4NAc [42,43]. The α-configuration of monosaccharides was confirmed by intraresidue H-1/H-2 cross-peaks for α-GalNAc and α-Rha4NAc residues in the NOESY spectrum (Figure 5). 

The ^1^H,^13^C HMBC spectrum showed correlations between H-2 of GalNAc and H-4 of all the Rha4NAc residues and carbonyl group signals in the range of δ_C_ 175.6–176.0 and between the latter and methyl proton signals at δ_H_ 2.05–2.06. This confirmed that all residues building the O-PS are *N*-acetylated. 

The relatively low-field positions of the signals for C-3 of Rha4NAc **A** and **D**, C-2 of Rha4NAc **B**, and C-4 of GalNAc **C** at δ 78.0, 74.3, 78.2 and 77.2, respectively, compared with their positions in the spectra of the corresponding non-substituted monosaccharides [42,43,44], were in agreement with the methylation analysis data and demonstrated the glycosylation pattern of the sugar residues. 

The NOESY spectrum (Figure 5) showed interresidue cross-peaks between residues **A**→**C**, **C**→**D**, and **D**→**A**. The correlations between the following anomeric protons and protons at the linkage carbons: Rha*p*4NAc **A** H-1/Gal*p*NAc **C** H-4 at δ 5.17/4.12; Gal*p*NAc **C** H-1/Rha*p*4NAc **D** H-3 at δ 5.04/3.94; and Rha*p*4NAc **D** H-1/Rha*p*4NAc **A** H-3 at δ 5.0/3.97 indicated that the polysaccharide O-PS1 is a heteropolymer with a trisaccharide repeating unit (Figure 5, Table 2). In turn, in the NOESY spectrum, intraresidue H-1/H-2 and interresidue H-1/H-5 correlations at δ 5.16/4.15 and δ 5.16/3.84, respectively, typical of α-(1→2)-linked sugars with the *manno* configuration, indicated that the Rha4NAc **B** residues constitute the other structurally different O-polysaccharide (O-PS2). 

**Figure 6:**
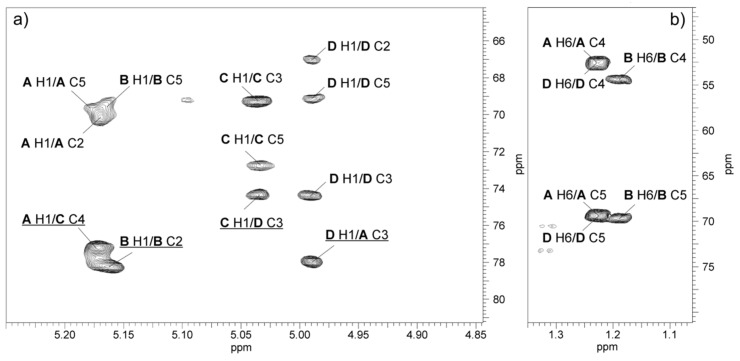
Regions of the ^1^H,^13^C HMBC spectrum of the O-PS of *A. hydrophila* JCM 3968, serotype O6. The maps show heteronuclear correlations for: (**a**) Anomeric protons, and (**b**) H-6 protons. Interresidue correlations between anomeric protons and carbons at the glycosidic linkages are underlined. Some other correlations H/C are depicted as well. Capital letters and Arabic numerals refer to protons or carbons in the sugar residues denoted as shown in Table 2.

The ^1^H,^13^C HMBC spectrum (Figure 6) displayed the following inter-glycosidic cross-peaks: **A** H-1/**C** C-4 at δ 5.17/77.2; **C** H-1/**D** H-3 at δ 5.04/74.3; and **D** H-1/**A** H-3 at δ 5.0/78.0 and revealed the sequence of sugars in the polysaccharide O-PS1. 

The analysis of the glycosylation effects on the ^13^C NMR chemical shifts confirmed the absolute configuration of Rha4NAc residues in the *A. hydrophila* JCM 3968 O-PS. The relatively small α-glycosylation effects of 3.0 ppm on C-1 of α-d-Gal*p*NAc and of 3.3 ppm on C-3 of Rha4NAc indicated different absolute configurations of the constituents in the disaccharide fragment α-d-Gal*p*NAc-(1→3)-α-l-Rha*p*4NAc **D** [45]. On the contrary, the relatively large α-effects of >8 ppm on C-1 of l-Rha4NAc **D** and ~7 ppm on C-3 in the α-l-Rha*p*4NAc-(1→3)-α-l-Rha*p*4NAc disaccharide fragment showed that the constituent monosaccharides have the same absolute configuration, i.e., Rha4NAc **A** has the l configuration (in the case of their different configurations, the effect on C-1 would be <5 ppm) [45]. 

In the disaccharide fragment α-d-Gal*p*NAc-(1→3)-α-l-Rha*p*4NAc **D**, the relatively large negative β-effect of >4 ppm on C-2 of Rha4NAc caused by its glycosylation at O-3 with α-d-Gal*p*NAc indicated that the linked monosaccharides have different absolute configurations, as the effect on C-2 in the case of the same configurations would be <1 ppm. The latter variant, i.e. a relatively small negative β-effect on C-2 of Rha4NAc caused by glycosylation at O-3, was observed in the α-l-Rha*p*4NAc-(1→3)-α-l-Rha*p*4NAc disaccharide fragment, where the linked monosaccharides have the same absolute configuration l [45]. 

In conclusion, the O-antigen of *A. hydrophila* JCM 3968, serogroup O6 consists of two structurally different O-polysaccharides. One of them (O-PS1) is a heteropolymer built up of trisaccharide repeating units composed of one α-d-Gal*p*NAc and two α-(1→3)-linked l-Rha*p*4NAc residues. The other polysaccharide, O-PS2, is an α-(1→2)-linked homopolymer of l-Rha4NAc. 

Based on the composition and methylation analyses as well as NMR data, the following structures of the O-polysaccharides were established:

O-PS1  →3)-α-l-Rha*p*4NAc-(1→4)-α-d-Gal*p*NAc-(1→3)-α-l-Rha*p*4NAc-(1→
**A**

**C**

**D**
O-PS2→2)-α-l-Rha*p*4NAc-(1→
**B**


The structure of the O-antigen is unique among O-polysaccharides of *Aeromonas* bacteria as well as other bacterial O-polysaccharides. To the best of our knowledge, this is the first work that shows the occurrence of l-perosamine as a component of bacterial O-polysaccharides. 

## 3. Discussion

Lipopolysaccharide (LPS, endotoxin), which is an important virulence factor of Gram-negative bacteria, belongs to molecules classified as PAMP (Pathogen Associated Molecular Patterns) being a powerful activator of the innate immune response during host invasion. The S-LPS species of smooth bacteria are composed of three domains: lipid A, a core oligosaccharide, and an O-specific polysaccharide. The lipid A moiety critically affects the biological activity of endotoxin by mediating the interaction of LPS with pattern recognition receptors, such as toll-like receptor 4 (TLR-4) on monocytes and macrophages, which eventually results in production of proinflammatory cytokines in response to the pathogen invasion [17,46,47,48]. In turn, the variable chemical structure of the O-polysaccharide portion determines the immunospecificity of individual bacterial strains and has become the basis for classification thereof to appropriate O serogroups. Serological typing has been used in epidemiological studies to define the routes of transmission or relationships with pathogenicity [49]. It is known that the specific O serogroups can be associated with certain clinical syndromes. Therefore, serological typing methods are valuable to relate characteristic serogroups, within many genera of Gram-negative bacteria e.g., *Escherichia coli*, *Salmonella*, *Proteu*s, *Citrobacter*, and *Aeromomas*, with virulence and several diseases. 

In this work, we established the structure of the O-antigen of *A. hydrophila* strain JCM 3968 (serogroup O6 reference strain) and found that it consisted of two different O-polysaccharides. The studied O-chain is unique among O-polysaccharides of *Aeromonas* bacteria as well as other bacterial O-polysaccharides (Bacterial Carbohydrate Structure Database: http://glyco.ac.ru/bcsdb). Moreover, another peculiar characteristic of the O-PS of *A. hydrophila* JCM3968, O6 that is worth emphasizing is the presence of 4-amino-4,6-dideoxymannose (Rha4N, perosamine), i.e., quite an unusual amino sugar, in the l-configuration. This is the first work that shows the occurrence of l-perosamine as a component of bacterial O-polysaccharides, whereas Rha4N in the d-configuration has been found as a sugar building some bacterial O-antigens. 

As reported here for the O-PS of *A. hydrophila* JCM 3978 serogroup O6, two structurally different polysaccharides, but containing d-perosamine, were identified in the O-polysaccharides of e.g., *Citrobacter gillenii* O9 (strain PCM 1537) [40], *Citrobacter youngae* O9 (strain PCM 1538) [43], and *Brucella melitensis* (strain 16M) [50,51]. One O-polysaccharide has been found to contain disaccharide, tetrasaccharide, or pentasaccharide repeating units composed of α1→2 and α1→3-linked residues of *N*-acetyl-d-perosamine or *N*-formyl-d-perosamine, as reported for the O-PS of *C. youngae* O9, *C. gillenii* O9, and *B. melitensis*, respectively. In turn, the other O-polysaccharide is an α1→2-linked homopolymer of 2-substituted d-perosamine residues *N*-acylated with various groups. Based on matrix-assisted laser-desorption/ionization mass spectrometry data, it was suggested that the O-chain elongation in the biosynthesis of both O-PS proceeds *via* sequential transfers of single sugar units to the nonreducing end of the growing polysaccharide chain. This model of biosynthesis requires the contribution of several distinct transferases for the same monosaccharide [52]. 

d-Rha4N was also detected as a sugar constituent of branched or linear bacterial O-antigens. The O-polysaccharides of *Brucella abortus* strain 1119-3 [53] and *Yersinia enterocolitica* O9 [54] are α-(1→2)-linked homopolymers of *N*-formyl-d-perosamine. In turn, homopolysaccharides of 4-amino-4,6-dideoxy-d-mannose decorated with (*S*)-2,4-dihydroxybutyryl or *N*-acetyl groups were found in the O-chains of *Vibrio cholerae* O1 and marine *V. cholerae* strain 487-85 (bio-serogroup Hakata), respectively [55,56]. 

Moreover, a Rha4N residue has been identified in heteropolymeric O-antigens composed of linear or branched repeating units. The linear tetrasaccharide repeating unit of the O-PS of *E. coli* O157:H7 [57], an enteric pathogen responsible for outbreaks of hemorrhagic colitis and hemolytic-uremic syndromes, consists of 2-substituted α-Rha*p*4NAc, 3-substituted α-Gal*p*NAc, 3-substituted α-Fuc*p*, and 4-substituted β-Glc*p*, and is structurally identical to the O-polysaccharides of *Citrobacter sedlakii* 6070 and *Salmonella enterica* O30 (group N) [58], giving also the basis for their serological cross-reactivity [52,59]. The branched O-antigen repeating units that shared a d-perosamine monosaccharide were also revealed in the trisaccharide O-units of *Xanthomonas cassavae* GSPB 2437 [60] and *Pseudomonas stutzeri* OX1 [61] and the heptasaccharide repeating unit of the main polysaccharide PS1 of *Caulobacter crescentus* JS1025 [62]. 

The chemical diversity of “somatic” O antigens is opposed to the structure of the LPS core region, especially the inner core part, which tends to be conserved within a genus or even family. The fact that the inner LPS core from distantly related bacteria shares structural features reflects the evolutionary relationship and the importance of this region in outer membrane integrity [63]. On the other hand, the outer core shows more structural diversity, as might be expected for a region exposed to the selective pressures of host responses, location of bacteriophage receptors, and environmental stress. 

A peculiar characteristic of the core oligosaccharide of the genus *Aeromonas* is the predominance of heptose residues over hexoses. The complete structure of the core OS of *A. hydrophila* strain AH-901, which is a rough mutant in a gene encoding mannose transferase, has been elucidated [36]. The core oligosaccharide is composed of four l,d-Hep and two d,d-Hep residues, β-Glc and α-GlcN, as well as one residue of α-3-deoxy-d-*manno*-oct-2-ulosonic acid at the reducing end. Additionally, one of the d,d-heptoses was non-stoichiometrically substituted with β-galactopyranose. No charged groups were reported except for a phosphate group at the 4-position of the Kdo. The structure of the LPS core of *A. hydrophila* strain AH-901 and *A. salmonicida* subsp. *salmonicida* (strains A449 and 80204) [36,64] shows great similarities in the inner LPS core and part of the outer LPS core. However, some differences have been found in the distal part of the outer core region (residues: l,d-Hep glycosylating at position C-6 of β-Glc, d-Gal, and d-GalNAc). Moreover, the structure was further elucidated through identification and characterization of three genomic regions in *A. hydrophila* AH-3 and *A. salmonicida* A450 involved in LPS core biosynthesis, which enabled the assignment of LPS core biosynthesis gene function [39,65]. 

Our latest findings have revealed that the core oligosaccharide with the predominance of heptose residues and the composition Hep_6_Hex_1_HexN_1_Kdo_1_P_1_ represents a structure shared by the LPS core part of strains belonging to the species *A. hydrophila* and *A. bestiarum* [28]. On the other hand, we have also reported that the core structure may vary to some extent within *Aeromonas* bacteria [27]. 

In this work, the phenol-soluble LPS isolated from *A. hydrophila* JCM strain 3968 has been structurally characterized. The compositional analysis and MALDI-TOF MS experiments have confirmed that the core oligosaccharide has a structure with the following composition: Hep_6_Hex_1_HexN_2_Ac_1_KdoP. Our study shows that the core oligosaccharide structure is slightly different from the structure previously reported for *A. hydrophila* AH-901 and *A. salmonicida* A450. The presence of both d,d- and l,d-Hep isomers makes the core OS studied here most similar to that of *A. hydrophila* AH-901 [36]. The detection of the 3-substituted GalNAc among permethylated alditol acetates of the dgPS suggests that the O-antigen in the LPS of *A. hydrophila* JCM 3968 is linked to the GalNAc residue in a manner similar to that in *A. salmonicida* subsp. *salmonicida* A450 [39]. In turn, in rough LPS glycoforms, the galactose residue appeared to be a terminal outer core sugar, similarly as has been established for the core of *A. hydrophila* strain AH-901 [36]. 

For both the LPS core oligosaccharides mentioned [36,39], the distal heptose residue (d,d-Hep or l,d-Hep), which substitutes β-Glc*p* at position C-6, is a crucial (outer core) sugar glycosylated at position C-4 by the monosaccharide (galactose or *N*-acetylgalactosamine) or the disaccharide β-d-Gal-(1→4)-β-d-GalNAc. The latter one, in turn, represents the site of attachment of the O-antigen polysaccharide. 

Recently, the studies performed by Merino and Tomas [66] revealed that the structure of the LPS core oligosaccharide from *A. salmonicida* subsp. *pectinolytica* (atypical *A. salmonicida* subspecies) is also consistent with the established core structure of *A. hydrophila* strain AH-3. Moreover, the genomic analyses of the *A. salmonicida* subsp. *pectinolytica* regions engaged in LPS core synthesis showed that the predicted gene functions were in agreement with the chemical structure. Nevertheless, as has been reported, a comparative *in silico* analysis of region 1 versus the whole sequenced genomes of 121 *Aeromonas* strains (belonging to the species *A. hydrophila*, *A. veronii*, *A. caviae*, *A. media*, and *Aeromonas* spp.) showed some different genes, which will eventually affect the structure of the LPS core oligosaccharide. 

The similarity in the LPS core oligosaccharide structure, especially in the inner core part of *Aeromonas* strains, which corresponds to the similarities in the organization of genomic regions, gives rationale to the recent assumptions [66], i.e., the LPS core genes, especially those structurally non-variable, could complement specific phylogenetic analyses. 

In conclusion, the O-PS of *A. hydrophila* JCM 3968 studied here contains a 4-acetamido-4-deoxy-l-perosamine, a monosaccharide that contributes to the uniqueness of the O-antigen and determines the specificity of serogroup O6. In future, immunochemical studies will be carried out to show structural similarities, but not identity, and serological relatedness of the O-PS of *A. hydrophila* JCM 3968, serogroup O6, to O-polysaccharides of *Aeromonas* strains isolated from fish during MAI/MAS outbreaks in Polish aquacultures. It is important to elucidate the serological and structural similarities and differences in O-chain polysaccharides in various serogroups and strains, which contribute to the immunospecificity of *Aeromonas*. 

## 4. Materials and Methods 

### 4.1. Bacterial Strain, Cultivation Conditions, and Isolation of LPS

*Aeromonas hydrophila* reference strain JCM (Japan Collection of Microorganisms) 3968, serogroup O6 was obtained from the collection of the Department of Fish Diseases, National Veterinary Research Institute (Puławy, Poland). The bacterium was cultivated with shaking (120 rpm) on tryptic soy broth (TSB) for 72 h at 28 °C. The cells were harvested by low speed centrifugation (8000*g*, 20 min). The recovered bacterial cell pellet was washed twice with 0.85% saline and once more with distilled water.

The bacterial cells (5 g dry mass) were digested with lysozyme, RNAse, and DNAse (24 h, 1 mg/g) and then with Proteinase K (36 h, 1 mg/g) in 50 mM phosphate buffer (pH 7.0) containing 5 mM MgCl_2_. The suspension was dialyzed against distilled water and freeze-dried. The digested cells were extracted three times with aq 45% phenol at 68 °C, [35] and the separated layers were dialyzed against tap and distilled water. LPS species recovered from the phenol phase were purified by ultracentrifugation at 105,000*g* and freeze-dried to give a yield of 4.8% of dry bacterial cell mass. 

### 4.2. Degradation of LPS and Isolation of O-polysaccharide

The phenol-soluble S-LPS (100 mg) was hydrolyzed with aq 2% acetic acid at 100 °C for 3 h, and lipid A precipitate was removed by centrifugation. The supernatant was concentrated and then fractionated by GPC on a column (1.8 × 80 cm) of Sephadex G-50 fine (Pharmacia, Sweden) using 1% acetic acid as the eluent and monitoring with a differential refractometer (Knauer, Berlin, Germany). The yield of the O-PS fraction was 31% of the LPS mass subjected to hydrolysis.

### 4.3. Chemical Analyses

For neutral and amino sugar analysis, the LPS and O-PS samples were hydrolyzed with 2 M CF_3_CO_2_H (100 °C, 4 h) or 10 M HCl for 30 min at 80 °C, respectively, and reduced with NaBD_4_; this was followed by acetylation with a 1:1 (v/v) mixture of acetic anhydride and pyridine (85 °C, 0.5 h). 

To release acidic sugar, LPS was dephosphorylated with 48% aqueous hydrofluoric acid, HF (4 °C, 18 h) and dried under vacuum over sodium hydroxide [37]. Methanolysis was performed with 1 M MeOH/HCl (85 °C, 1 h), and the sample was extracted twice with hexane. The methanol layer was concentrated and the residue was dried and acetylated. The monosaccharides were identified as alditol and aminoalditol acetates [67] as well as acetylated methyl glycosides by GLC-MS on an Agilent Technologies 7890A (Agilent Technologies, Wilmington, DE, USA) gas chromatograph connected to a 5975C MSD (inert XL EI/CI, Agilent Technologies, Wilmington, DE, USA) detector. The chromatograph was equipped with an HP-5MS capillary column (Agilent Technologies, 30 m × 0.25 mm, flow rate of 1 mL/min, He as carrier gas), and a temperature gradient of 150 °C (5 min) to 310 °C at 5 °C min^−1^ was applied. For determination of the absolute configuration [41], the O-PS was subjected to 2-octanolysis (300 μL (*S*)-(+)-2-octanol or (*SR*)-(±)-2-octanol and 20 μL acetyl chloride, 100 °C, 3 h); the products were acetylated and analyzed by GLC-MS as above. A sample from the polysaccharide of *Citrobacter gillenii* O9a,9b [40] was used as the reference standard of d-Rha4N (d-perosamine). 

Methylation of the O-PS (1.0 mg) was carried out with methyl iodide or CD_3_ iodide in dimethyl sulfoxide in the presence of powdered sodium hydroxide [68]. The products were recovered by extraction with chloroform/water (1:1, v/v), hydrolyzed with 10 M HCl for 30 min at 80 °C, *N*-acetylated, and reduced with NaBD_4_. The partially methylated alditol acetates derivatives were analyzed by GLC-MS. Methylation of the dgPS (1.5 mg) and the *N*-acetylated core oligosaccharide (1 mg) were carried out with the use of methyl iodide [68]. The products were hydrolyzed with 2 M CF_3_CO_2_H (4 h at 100 °C), *N*-acetylated, reduced, acetylated and analyzed by GLC-MS as above. 

For fatty acid analysis, a sample of the lipid A (1 mg) was subjected to methanolysis in 2 M methanolic HCl (85 °C, 12 h). The resulting fatty acid methyl esters were extracted with hexane and converted to their *O*-trimethylsilyl (*O*-TMS) derivatives, as described [69,70]. The methanol layer containing methyl glycosides was dried and acetylated with a pyridine-acetic anhydride mixture. The fatty acid derivatives and acetylated methyl glycosides were analyzed by GLC-MS as above. 

### 4.4. NMR Spectroscopy

An O-PS sample was deuterium-exchanged by freeze-drying from D_2_O and then examined in 99.98% D_2_O using acetone as an internal standard (δ_H_ 2.225, δ_C_ 31.45). 1D and 2D NMR spectra were recorded at 32 °C on the 500 MHz NMR Varian Unity Inova instrument (Varian Associates, Palo Alto, CA, USA) using the Varian software Vnmrj V. 4.2 rev. (Agilent Technologies, Santa Clara, CA, USA). The following homonuclear and heteronuclear shift-correlated two-dimensional experiments were conducted for signal assignments and determination of the sugar sequence: ^1^H,^1^H DQF-COSY, ^1^H,^1^H TOCSY,^1^H,^1^H NOESY, ^1^H,^13^C HSQC, and ^1^H,^13^C HMBC. The mixing time was set to 100 and 200 ms in the TOCSY and NOESY experiments, respectively. The ^1^H,^13^C HSQC experiment with CRISIS based multiplicity editing was optimized for a coupling constant of 146 Hz. The heteronuclear multiple-bond correlation (HMBC) experiment was optimized for *J*_H,C_ = 7 and 5 Hz, with 2-step low-pass filter 130 and 165 Hz to suppress one-bond correlations. 

### 4.5. MALDI-TOF Mass Spectrometry (MS)

LPS was analyzed with matrix-assisted laser desorption/ionization time-of flight (MALDI-TOF) mass spectrometry (MS) using a Waters SYNAPT G2-*Si* HDMS instrument (Waters Corporation, Milford, MA, USA) equipped with a 1 kHz Nd:YAG laser system. Acquisition of the data was performed using MassLynx software version 4.1 SCN916 (Waters Corporation, Wilmslow, United Kingdom). Mass spectra were assigned with a multi-point external calibration using red phosphorous (Sigma-Aldrich, St. Louis, MO, USA) and recorded in the negative ion mode. An LPS sample (at a concentration of 10 µg/µL) was suspended in a water/methanol (1:1, v/v) solution containing 5 mM EDTA and then dissolved by ultrasonication. After desalting with some grains of cation exchange beads (Dowex 50WX8-200; Sigma-Aldrich, St. Louis, MO, USA), one microliter of the sample was transferred onto a well plate covered with a thin matrix film and allowed to dry at room temperature. The matrix solution was prepared from 2’,4’,6’-trihydroxyacetophenone (THAP) (200 mg/mL in methanol) mixed with nitrocellulose (15 mg/mL) suspended in 2-propanol/acetone (1:1, v/v) in proportion of 4:1 (v/v), according to the published method [71]. 

### 4.6. SDS-PAGE

LPS preparations were separated in 12.5% SDS-Tricine polyacrylamide electrophoresis gel and bands were visualized by silver staining after oxidation with periodate according to the published method [72]. 

## Figures and Tables

**Table 1:** Composition of the main species present in the negative ion MALDI-TOF mass spectra of the LPS (**a**) and the core oligosaccharide (**b**) of *A. hydrophila* JCM 3968.

[M − H]^‒^ Observed	[M − H] ^‒^ Calculated	M Monoisotopic	Composition
1570.012	1569.946	1570.953	HexN_2_P_2_[14:0(3-OH)]_2_[*i*15:0(3-OH)](12:0)_2_
1698.172	1698.162	1699.169	HexN_2_P_1_[14:0(3-OH)]_3_[*i*15:0(3-OH)](12:0)_2_
1768.187	1768.181	1769.188	HexN_2_P_2_[14:0(3-OH)]_4_(12:0)_2_
1796.230	1796.139	1797.146	HexN_2_P_2_[14:0(3-OH)]_3_[*i*15:0(3-OH)](12:0)_2_
1891.587	1891.598	1892.605	[Hep_6_Hex_1_HexN_2_Kdo*_anh_*P]-COO
1935.611	1935.588	1936.595	Hep_6_Hex_1_HexN_2_Kdo*_anh_*P
1977.615	1977.599	1978.606	Hep_6_Hex_1_HexN_2_Ac_1_Kdo*_anh_*P
2555.127	2554.846	2555.853	[6dHexNAc_2_HexN_3_Ac_2_Hep_6_Hex_1_Kdo*_anh_*P]-H_2_O
1664.534	1664.542	1665.550	Hep_5_Hex_2_HexN_1_Kdo*_anh_*
1694.541	1694.553	1695.560	Hep_6_Hex_1_HexN_1_Kdo*_anh_*
1856.592	1856.605	1857.613	Hep_6_Hex_2_HexN_1_Kdo*_anh_*

**Table 2:** ^1^H (500 MHz) and ^13^C NMR (125 MHz) data (δ, ppm) for the O-PS of *A. hydrophila* strain JCM 3968.

Sugar Residue		Chemical Shifts (δ, ppm)					
		H-1 C-1	H-2 C-2	H-3 C-3	H-4 C-4	H-5 C-5	H-6 C-6
→3)-α-l-Rha*p*4NAc-(1→	**A**	5.17	4.20	3.97	3.97	3.92	1.23
		103.0	70.3	78.0	52.5	69.4	18.0
→2)-α-l-Rha*p*4NAc-(1→	**B**	5.16	4.15	4.05	3.91	3.84	1.19
		101.7	78.2	69.2	54.3	69.6	18.0
→4)-α-d-Gal*p*NAc-(1→	**C**	5.04	4.26	3.97	4.12	3.90	3.74, 3.81
		95.0	50.9	69.2	77.2	72.7	62.2
→3)-α-l-Rha*p*4NAc-(1→	**D**	5.00	3.91	3.94	3.98	3.92	1.23
		103.0	67.0	74.3	52.3	69.4	18.0

Chemical shifts for NAc are δ_H_ 2.05–2.06 and δ_C_ 23.3–23.4/175.6–176.0.

## References

[1] Janda J.M., Duffy P.S. (1988). Mesophilic aeromonads in human diseases: Current taxonomy, laboratory infection and infectious diseases spectrum. Rev. Infect. Dis..

[2] Janda J.M. (1991). Recent advances in the study of the taxonomy, pathogenicity and infectious syndromes with the genus *Aeromonas*. Clin. Microbiol. Rev..

[3] Nawaz M., Khan S.A., Khan A.A., Sung K., Tran Q., Kerdahi K., Steele R. (2010). Detection and characterization of virulence genes and integrons in *Aeromonas veronii* isolated from catfish. Food Microbiol..

[4] Araujo R.M., Arribas R.M., Pares R. (1991). Distribution of *Aeromonas* species in waters with different levels of pollution. J. Appl. Bacteriol..

[5] Janda J.M., Abbott S. (2010). The genus *Aeromonas*: Taxonomy, pathogenicity, and infection. Clin. Microbiol. Rev..

[6] Holmberg S.D., Schell W.L., Fanning G.R., Wachsmuth I.K., Blake P.A., Brenner D.J., Farmer J.J. (1986). *Aeromonas* intestinal infections in the United States. Ann. Int. Med..

[7] Ali A., Carnahan A.M., Altwegg M., Luthy-Hottenstein J., Joseph S.W. (1996). *Aeromonas bestiarum* sp. nov. (formerly genomospecies DNA group 2 *A. hydrophila*), a new species isolated from non human sources. Med. Microbiol. Lett..

[8] Kahajanchi B.K., Fadl A.A., Borchardt M.A., Berg R.L., Horneman A.J., Stemper M.E., Joseph S.W., Moyer N.P., Sha J., Chopra A.K. (2010). Distribution of virulence factors and molecular fingerprinting of *Aeromonas* species isolates from water and clinical samples: Suggestive evidence of water-to-human transmission. Appl. Environ. Microbiol..

[9] Figueras M.J. (2005). Clinical relevance of *Aeromonas* sM503. Rev. Clin. Microbiol..

[10] Tomas J.M. (2012). The Main *Aeromonas* Pathogenic Factors. ISRN Microbiology..

[11] Dooley J.S.G., Lallier R., Shaw D.H., Trust T.J. (1985). Electrophoretic and immunochemical analyses of the lipopolysaccharides from various strains of *Aeromonas hydrophila*. J. Bacteriol..

[12] Merino S., Rubires X., Aguillar A., Guillot J.F., Tomas J.M. (1996). The role of the O-antigen lipopolysaccharide on the colonization in vivo of the germfree chicken gut by *Aeromonas hydrophila* serogroup O:34. Microb. Pathog..

[13] Aguilar A., Merino S., Rubires X., Tomas J. (1997). Influence of osmolarity on lipopolysaccharides and virulence of *Aeromonas hydrophila* serotype O:34 strains grown at 37 °C. Infect. Immun..

[14] Rabaan A.A., Gryllos I., Tomas J.M., Shaw J.G. (2001). Motility and polar flagellum are required for *Aeromonas caviae* adherence to HEp-2 cells. Infect. Immun..

[15] Garduño R.A., Moore A.R., Oliver G., Lizama A.L., Garduño E., Kay W.W. (2000). Host cell invasion and intracellular resistance by *Aeromonas salmonicida*: Role of the *S*-layer. J. Clin. Microbiol..

[16] Asha A., Nayak D.K., Shankar K.M., Mohan C.V. (2004). Antigen expression in biofilm cells of *Aeromonas hydrophila* employed in oral vaccination of fish. Fish Shellfish Immun..

[17] Caroff M., Karibian D. (2003). Structure of bacterial lipopolysaccharides. Carbohydr. Res..

[18] Nazarenko E.L., Crawford R.J., Iwanowa E.P. (2011). The structural diversity of carbohydrate antigens of selected Gram-negative marine bacteria. Mar. Drugs.

[19] Sakazaki R., Shimada T. (1984). *O*-Serogrouping for mesophilic *Aeromonas* strains. Jpn. J. Med. Sci. Biol..

[20] Thomas L.V., Gross R.J., Cheasty T., Rowe B. (1990). Extended serogrouping scheme for motile, mesophilic *Aeromonas* species. J. Clin. Microbiol..

[21] Esteve C., Alcaide E., Canals R., Merino S., Blasco D., Figueras M.J., Tomas J.M. (2004). Pathogenic *Aeromonas hydrophila* serogroup O:14 and O:81 strains with S-layer. Appl. Environ. Microbiol..

[22] Kozinska A., Pekala A. (2010). Serotyping of *Aeromonas* species isolated from Polish fish farms in relation to species and virulence phenotype of the bacteria. Bull. Vet. Inst. Pulawy.

[23] Show D.H., Squires M.J. (1984). O-antigen structure in a virulent strain *Aeromonas hydrophila*. FEMS Microbiol. Lett..

[24] Merino S., Canals R., Knirel Y.A., Tomas J.M. (2015). Molecular and chemical analysis of the lipopolysaccharide from *Aeromonas hydrophila* strain AH-1 (Serotype O11). Mar. Drugs.

[25] Pieretti G., Carillo S., Lanzetta R., Parrilli M., Merino S., Tomas J.M., Corsaro M.M. (2011). Structural determination of the O-specific polysaccharide from *Aeromonas hydrophila* strain A19 (serogroup O:14) with S-layer. Carbohydr. Res..

[26] Knirel Y.A., Shashkov A.S., Senchenkova S.N., Merino S., Tomas J.M. (2002). Structure of the O-specific polysaccharide of *Aeromonas hydrophila* O:34; a case of random *O*-acetylation of 6-deoxy-L-talose. Carbohydr. Res..

[27] Turska-Szewczuk A., Duda K.A., Schwudke D., Pekala A., Kozinska A., Holst O. (2014). Structural studies of the lipopolysaccharide from the fish pathogen, *Aeromonas veronii* strain Bs19, serotype O16. Mar. Drugs.

[28] Turska-Szewczuk A., Lindner B., Komaniecka I., Kozinska A., Pekala A., Choma A., Holst O. (2013). Structural and immunochemical studies of the lipopolysaccharide from the fish pathogen, *Aeromonas bestiarum* strain K296, serotype O18. Mar. Drugs.

[29] Wang Z., Vinogradov E., Larocque S., Harrison B.A., Li J., Altman E. (2005). Structural and serological characterization of the O-chain polysaccharide of *Aeromonas salmonicida* strains A449, 80204 and 80204–1. Carbohydr. Res..

[30] Wang Z., Liu X., Dacanay A., Harrison B.A., Fast M., Colquhoun D.J., Lund V., Brown L.L., Li J., Altman E. (2007). Carbohydrate analysis and serological classification of typical and atypical isolates of *Aeromonas salmonicida*: a rationale for the lipopolysaccharide-based classification of *A. salmonicida*. Fish Shellfish Immun..

[31] Wang Z., Liu X., Li J., Altman E. (2008). Structural characterization of the O-chain polysaccharide of *Aeromonas caviae* ATCC 15468 lipopolysaccharide. Carbohydr. Res..

[32] Turska-Szewczuk A., Pietras H., Duda K.A., Kozińska A., Pękala A., Holst O. (2015). Structure of the O-specific polysaccharide from the lipopolysaccharide of *Aeromonas sobria* strain Pt312. Carbohydr. Res..

[33] Turska-Szewczuk A., Kozinska A., Russa R., Holst O. (2010). The structure of the O-specific polysaccharide from the lipopolysaccharide of *Aeromonas bestiarum* strain 207. Carbohydr. Res..

[34] Turska-Szewczuk A., Guz L., Lindner B., Pietras H., Russa R., Holst O. (2011). Structural characterization of the *O*-specific polysaccharide from the lipopolysaccharide of fish pathogen *Aeromonas bestiarum* strain P1S. Carbohydr. Res..

[35] Westphal O., Jann K. (1965). Bacterial lipopolysaccharide. Extraction with phenol-water and further applications of the procedure. Meth. Carbohydr. Chem..

[36] Knirel Y.A., Vinogradov E., Jimenez N., Merino S., Tomas J.M. (2004). Structural studies on the R-type lipopolysaccharide of *Aeromonas hydrophila*. Carbohydr. Res..

[37] Pieretti G., Corsaro M.M., Lanzetta R., Parrilli M., Nicolaus B., Gambacorta A., Lindner B., Holst O. (2008). Structural characterization of the core region of the lipopolysaccharide from the haloalkaliphilic *Halomonas pantelleriensis*: identification of the biological O-antigen repeating unit. Eur. J. Org. Chem..

[38] Domon B., Costello C.E. (1988). A systamatic nomenclature for carbohydrate fragmentations in FAB MS/MS spectra of glycoconjugates. Glycoconj. J..

[39] Jimenez N., Lacasta A., Vilches S., Reyes M., Vazquez J., Aquillini E., Merino S., Regue M., Tomas J.M. (2009). Genetics and proteomics of *Aeromonas salmonicida* lipopolysaccharide core biosynthesis. J. Bacteriol..

[40] Lipiński T., Zatonsky G.V., Kocharova N.A., Jaquinod M., Forest E., Shashkov A.S., Gamian A., Knirel Y.A. (2002). Structures of two O-chain polysaccharides of *Citrobacter gillenii* O9a,9b lipopolysaccharide. A new homopolymer of 4-amino-4,6-dideoxy-D-mannose (perosamine). Eur. J. Biochem..

[41] Leontein K., Lindberg B., Lönngren J. (1978). Assignment of absolute configuration of sugars by GLC of their acetylated glycosides formed from chiral alcohols. Carbohydr. Res..

[42] Lipkind G.M., Shashkov A.S., Knirel Y.A., Vinogradov E.V., Kochetkov N.K. (1988). A computer-assisted structural analysis of regular polysaccharides on the basis of 13C-n.m.r. data. Carbohydr. Res..

[43] Ovchinnikova O.G., Kocharova N.A., Katzenellenbogen E., Zatonsky G.V., Shashkov A.S., Knirel Y.A., Lipiński T., Gamian A. (2004). Structures of two O-polysaccharides of the lipopolysaccharide of *Citrobacter youngae* PCM 1538 (serogroup O9). Carbohydr. Res..

[44] Jansson P.E., Kenne L., Widmalm G. (1989). Computer-assisted structural analysis of polysaccharides with an extended version of CASPER using ^1^H- and ^13^C-NMR data. Carbohydr. Res..

[45] Shashkov A.S., Lipkind G.M., Knirel Y.A., Kochetkov N.K. (1988). Stereochemical factors determining the effects of glycosylation on the ^13^C NMR shifts in carbohydrates. Magn. Reson. Chem..

[46] Mogensen T.H. (2009). Pathogen recognition and inflammatory signaling in innate immune defenses. Clin. Microbiol. Rev..

[47] Kaszowska M., Wojcik M., Sudnienko J., Lugowski C., Lukasiewicz J. (2017). Structure-activity relationship of *Plesiomonas shigelloides* lipid A to the production of TNF-α, IL-1β, and IL-6 by human and murine macrophages. Front. Immunol..

[48] Karaś M.A., Turska-Szewczuk A., Janczarek M., Szuster-Ciesielska A. (2019). Glycoconjugates of Gram-negative bacteria and parasitic protozoa – are they similar in orchestrating the innate immune response?. Innate Immun..

[49] Arteaga Garibay R.I., Aguilera-Arreola M.G., Navarro Ocana A., Giono Cerezo S., Sanchez Mendoza M., Molina Lopez J., Eslava Campos C., Cravioto A., Castro-Escarpulli G. (2006). Serogroups, K1 antigen, and antimicrobial resistance patterns of *Aeromonas* spp. strains isolated from different sources in Mexico. Mem. Inst. Oswaldo Cruz.

[50] Bundle D.R., Cherwonogrodzky J.W., Perry M.B. (1987). The structure of the lipopolysaccharide O-chain (M antigen) and polysaccharide B produced by *Brucella melitensis* 16M. FEBS Lett..

[51] Kubler-Kielb J., Vinogradov E. (2013). Reinvestigation of the structure of *Brucella* O-antigens. Carbohydr. Res..

[52] Knirel Y.A., Kocharova N.A., Bystrova O.V., Katzenellenbogen E., Gamian A. (2002). Structures and serology of the O-specific polysaccharides of bacteria of the genus *Citrobacter*. Arch. Immunol. Ther. Exp..

[53] Caroff M., Bundle D.R., Perry M.B., Cherwonogrodzky J.W., Dunkan J.R. (1984). Antigenic S-type lipopolysaccharide of *Brucella abortus* 1119–3. Infect. Immun..

[54] Caroff M., Bundle D.R., Perry M.B. (1984). Structure of the O-chain of the phenol-phase soluble cellular lipopolysaccharide of *Yersinia enterocolitica* serotype O:9. Eur. J. Biochem..

[55] Kenne L., Lindberg B., Unger P., Gustafsson B., Holme T. (1982). Structural studies of the *Vibrio cholerae* O-antigen. Carbohydr. Res..

[56] Haishima Y., Kondo S., Hisatsune K. (1990). The occurrence of α(1→2) linked *N*-acetylperosamine-homopolymer in lipopolysaccharides of non-O1 *Vibrio cholerae* possessing an antigenic factor in common with O1 *V. cholerae*. Microbiol. Immunol..

[57] Perry M.B., MacLean L., Griffith D.W. (1986). Structure of the O-chain polysaccharide of the phenol-phase soluble lipopolysaccharide of *Escherichia coli* O:157:H7. Biochem. Cell Biol..

[58] Bundle D.R., Gerken M., Perry M.B. (1986). Two-dimensional nuclear magnetic resonance at 500 MHz: the structural elucidation of a *Salmonella* serogroup N polysaccharide antigen. Can. J. Chem..

[59] Vinogradov E., Conlan J.W., Perry M.B. (1998). Serological cross-reaction between the lipopolysaccharide O-polysaccharide antigens of *Escherichia coli* O157:H7 and strains of *Citrobacter freundii* and *Citrobacter sedlakii*. FEMS Microbiol. Lett..

[60] Senchenkova S.N., Shashkov A.S., Knirel Y.A., Wydra K., Witt F., Mavridis A., Rudolph K. (2004). Structure of two O-polysaccharide of *Xanthomonas cassavae* GSPB 2437. Carbohydr. Res..

[61] Leone S., Izzo V., Lanzetta R., Molinaro A., Parrilli M., Di Donato A. (2005). The structure of the O-polysaccharide from *Pseudomonas stutzeri* OX1 containing two different 4-acylamido-4,6-dideoxy-residues, tomosamine and perosamine. Carbohydr. Res..

[62] Jones M.D., Vinogradov E., Nomellini J.F., Smit J. (2015). The core and O-polysaccharide structure of the *Caulobacter crescentus* lipopolysaccharide. Carbohydr. Res..

[63] Raetz C.R.H., Whitfield C. (2002). Lipopolysaccharide endotoxins. Annu. Rev. Biochem..

[64] Wang Z., Li J., Vinogradov E., Altman E. (2006). Structural studies of the core region of *Aeromonas salmonicida* subsp. *salmonicida* lipopolysaccharide. Carbohydr. Res..

[65] Jimenez N., Canals R., Lacasta A., Kondakova A., Lindner B., Knirel Y.A., Merino S., Regue M., Tomas J.M. (2008). Molecular analysis of three *Aeromonas hydrophila* AH-3 (Serotype O34) lipopolysaccharide core biosynthesis gene clusters. J. Bacteriol..

[66] Merino S., Tomas J.M. (2016). The *Aeromonas salmonicida* lipopolysaccharide core from different subspecies: the unusual subsp. *pectinolytica*. Front. Microbiol..

[67] Russa R., Urbanik-Sypniewska T., Lindström K., Mayer H. (1995). Chemical characterization of two lipopolysaccharide species isolated from *Rhizobium loti* NZP2213. Arch. Microbiol..

[68] Ciucanu I., Kerek F. (1984). A simple and rapid method for the permethylation of carbohydrates. Carbohydr. Res..

[69] Komaniecka I., Choma A., Lindner B., Holst O. (2010). The structure of a novel lipid A from the lipopolysaccharide of *Bradyrhizobium elkanii* containing three mannose units in the backbone. Chem. Eur. J..

[70] Pieretti G., Corsaro M.M., Lanzetta R., Parrilli M., Vilches S., Merino S., Tomas J.M. (2009). Structure of the core region from the lipopolysaccharide of *Plesiomonas shigelloides* strain 302–73 (serotype O1). Eur. J. Org. Chem..

[71] Silipo A., Molinaro A., Sturiale L., Dow J.M., Erbs G., Lanzetta R., Newman M.A., Parrilli M. (2005). The elicitation of plant innate immunity by lipooligosaccharide of *Xanthomonas campestris*. J. Biol. Chem..

[72] Tsai C.M., Frasch C.E. (1982). A sensitive silver stain for detecting lipopolysaccharides in polyacrylamide gels. Anal. Biochem..

